# Web-Based Interventions to Help Australian Adults Address Depression, Anxiety, Suicidal Ideation, and General Mental Well-being: Scoping Review

**DOI:** 10.2196/31018

**Published:** 2022-02-08

**Authors:** Gemma Skaczkowski, Shannen van der Kruk, Sophie Loxton, Donna Hughes-Barton, Cate Howell, Deborah Turnbull, Neil Jensen, Matthew Smout, Kate Gunn

**Affiliations:** 1 Department of Rural Health, Allied Health and Human Performance University of South Australia Adelaide Australia; 2 Australian Medical Placements Health Education and Training Adelaide Australia; 3 Torrens University Adelaide Australia; 4 School of Psychology The University of Adelaide Adelaide Australia; 5 Freemasons Centre for Male Health and Wellbeing Adelaide Australia; 6 Justice and Society University of South Australia Adelaide Australia

**Keywords:** web-based interventions, depression, anxiety, suicide, well-being, mental health, technology, access to health care

## Abstract

**Background:**

A large number of Australians experience mental health challenges at some point in their lives. However, in many parts of Australia, the wait times to see general practitioners and mental health professionals can be lengthy. With increasing internet use across Australia, web-based interventions may help increase access to timely mental health care. As a result, this is an area of increasing research interest, and the number of publicly available web-based interventions is growing. However, it can be confusing for clinicians and consumers to know the resources that are evidence-based and best meet their needs.

**Objective:**

This study aims to scope out the range of web-based mental health interventions that address depression, anxiety, suicidal ideation, or general mental well-being and are freely available to Australian adults, along with their impact, acceptability, therapeutic approach, and key features.

**Methods:**

The PRISMA (Preferred Reporting Items for Systematic Reviews and Meta-Analyses) guidelines for scoping reviews (PRISMA-ScR [PRISMA extension for Scoping Reviews]) guided the review process. Keywords for the search were depression, anxiety, suicide, and well-being. The search was conducted using Google as well as the key intervention databases Beacon, Head to Health, and e-Mental Health in Practice. Interventions were deemed eligible if they targeted depression, anxiety, suicidal ideation, or general mental well-being (eg, resilience) in adults; and were web-based, written in English, interactive, free, and publicly available. They also had to be guided by an evidence-based therapeutic approach.

**Results:**

Overall, 52 eligible programs were identified, of which 9 (17%) addressed depression, 15 (29%) addressed anxiety, 13 (25%) addressed general mental well-being, and 13 (25%) addressed multiple issues. Only 4% (2/52) addressed distress in the form of suicidal ideation. The most common therapeutic approach was cognitive behavioral therapy. Half of the programs guided users through exercises in a set sequence, and most programs enabled users to log in and complete the activities on their own without professional support. Just over half of the programs had been evaluated for their effectiveness in reducing symptoms, and 11% (6/52) were being evaluated at the time of writing. Program evaluation scores ranged from 44% to 100%, with a total average score of 85%.

**Conclusions:**

There are numerous web-based programs for depression, anxiety, suicidal ideation, and general well-being, which are freely and publicly available in Australia. However, identified gaps include a lack of available web-based interventions for culturally and linguistically diverse populations and programs that use newer therapeutic approaches such as acceptance and commitment therapy and dialectical behavior therapy. Despite most programs included in this review being of good quality, clinicians and consumers should pay careful attention when selecting which program to recommend and use, as variations in the levels of acceptability and impact of publicly available programs do exist.

## Introduction

### Background

Approximately half of the Australian population experiences a common mental disorder (eg, anxiety and depression) at some point in their lives [1]. Mental and substance use disorders are the fourth highest contributor to total disease burden after cancer, cardiovascular disease, and musculoskeletal conditions [2], whereas suicide is the leading cause of death among young people aged between 15 and 44 years and the fourth leading cause of death in those aged between 45 and 64 years [2]. Despite these alarming statistics, only one-third of people experiencing mental illness will access support services [3]. The reasons for this may be structural (eg, availability of suitable services), knowledge-related (eg, limited awareness of the benefits of accessing mental health services), or attitudinal (eg, stigma and stoicism).

When people do seek help for mental health issues, 71% will contact their general practitioner (GP) at the first instance [3]. GPs play a crucial role in suicide prevention; approximately 45% of people who die by suicide have had contact with primary care in the preceding month and 77% in the preceding year [4]. However, GPs report the lack of tools needed to administer psychological care, including a lack of time, resources, and confidence [5]. Although GPs and their patients increasingly value psychological support, a shortage of professionals and time on wait lists, averaging between 2 and 6 months, is both frustrating and distressing for GPs and their patients.

There is a pressing need to bridge the gap between primary health and mental health care, particularly for Australians facing greater difficulties in accessing mental health care and who are at particular risk of suicide, such as those in rural areas. Fortunately, Australians are increasingly willing to use the internet to search for health-related information [6], with at least 86% of Australian households now having access to the internet [7]. Therefore, web-based resources and interventions may supplement face-to-face services, providing support to people as they wait for appointments [8] and new avenues to reach those who face structural or attitudinal barriers to accessing face-to-face mental health services [9]. Web-based resources have the advantage of enabling consumers’ anonymity and access at any time of the day or night, from the privacy and convenience of their own homes [8].

Across the globe, web-based interventions have been shown to effectively reduce the severity of symptoms of depression [10], anxiety [11], and social anxiety [12] and increase mental health literacy [13] and the ability to recognize, accept, deal with, and help prevent mental health issues [14]. The quality of this evidence is high, with a number of randomized controlled trials (RCTs; eg, the studies by McDermott et al [10], Powell et al [12], and Kiropoulos et al [13]) and systematic reviews (eg, the studies by Renton et al [15] and Ashford et al [16]) illustrating their value. Participation has also been associated with decreases in personal stigmatizing attitudes toward depression in the mainstream population [17] and among immigrants [13], as well as decreases in stigmatizing attitudes toward suicide [18] and help seeking [19]. Importantly, web-based interventions have been identified as acceptable in difficult-to-reach populations such as farmers [20], young people [21], and culturally and linguistically diverse groups [22,23]. They can be successfully integrated into routine primary care [24,25], and many Australian GPs support the notion of aiding their patients’ mental well-being through these resources [26]. In addition, web-based interventions have been found to be cost-effective for development and delivery; many are offered for free to consumers [8]. However, with the plethora of interventions emerging on the internet [27], there is a need for reliable and clear recommendations to help clinicians and consumers select web-based interventions that are based on evidence-based therapeutic approaches and meet their needs. The e-Mental Health in Practice (eMHPrac) project, funded by the Australian Government, has a range of resources to help Australian health practitioners find digital mental health programs for use with their patients [28]. This provides an excellent quick reference guide for clinicians. However, it does not outline the types of therapeutic approaches and tools used or indicate whether resources are evidence-based.

A total of 2 existing international scoping reviews summarize the characteristics of web-based interventions available for the treatment of depression in Canada in 2014 [15] and anxiety in the United Kingdom in 2016 [16]. The review of programs for depression by Renton et al [15] identified 32 programs, with only 12 having published evidence of efficacy. Furthermore, the review of programs for anxiety by Ashford et al [16] identified 34 programs, with only 17 having published evidence of efficacy. Given the rapid creation of web-based mental health resources in recent years [27] and the tendency for web-based mental health programs to change, arise, and disappear with the provision or withdrawal of funding, there is a need for updated information on interventions that consumers and clinicians are likely to come across via search engines in the Australian context. Of particular benefit to clinicians would be a focus on free and publicly available interventions, based on evidence-based therapeutic approaches, that Australians can immediately find or be referred to. Clinicians have also indicated that it would be useful to include web-based interventions for adults seeking help for *general mental well-being* and *suicidal ideation*, which are issues commonly encountered in clinical practice but not covered by past reviews. We note that many reviews of the evidence for web-based interventions for specific disorders (eg, depression [29-31]) and for specific populations (eg, adolescents and young adults [32,33]) have been undertaken in recent years. Although these are helpful for researchers and intervention developers, they are less likely to meet the practical information needs of consumers and clinicians, as many of the interventions contained in the reviews are not available outside research settings, and others that are publicly accessible, have not been evaluated and therefore are not included in the reviews.

### Objectives

The purpose of this research is to identify the scope of and evidence for web-based mental health interventions addressing depression, anxiety, suicidal ideation, or general mental well-being that are currently available for free to Australian adults, and in doing so, develop a quick reference guide for GPs and mental health clinicians. It seeks to answer the following questions [34]:

1. What web-based interventions addressing depression, anxiety, suicidal ideation, or general mental well-being are currently freely available (ie, at no cost and open to Australians) in Australia?
2. What are the key gaps?
3. What are the characteristics, including evidence of impact, credibility, and accessibility, of the interventions that are available?

## Methods

This scoping review was conducted in accordance with the PRISMA-ScR (Preferred Reporting Items for Systematic Reviews and Meta-Analyses extension for Scoping Reviews) [35]. The study protocol was registered in the Open Science Framework [36].

### Eligibility Criteria

Eligible interventions were those that (1) targeted depression, anxiety, suicidal ideation, or general mental well-being (eg, resilience and stress); (2) were web-based; (3) had an interactive component (ie, the delivery of content was not entirely in a passive manner); (4) were designed for adults; (5) were free (ie, at no cost) and publicly available (including via registration, application, or referral from a health care professional); (6) were available in English; and (7) were based on an evidence-based therapeutic approach.

Interventions were excluded if (1) they were targeted at consumers with a primary general medical condition (eg, survivors of cancer); (2) solely provided reading materials on one of the inclusion outcomes (ie, psychoeducation) or only offered a single tool (eg, diary-keeping, mood-monitoring, and meditation) without relevant psychoeducation or explanation of a broader evidence-based therapeutic approach; (3) were targeted at health professionals for training purposes; (4) were part of a research project *that restricted access* or limited program availability; or (5) included classes that had to be attended in person.

### Search Strategy

A search was conducted in October 2020 for key terms using Google (Australian version). As the previously published scoping review on web-based interventions for depression only found 1 additional website when searching Yahoo and Bing [15], only Google was searched for this review. A total of 4 searches were conducted by searching the following keywords: *depression*, *anxiety*, *suicide*, and *wellbeing*. A search log was kept to record the number of hits for each search, as well as the number of websites that were included or excluded (Multimedia Appendix 1). The search was terminated for the terms after searching the first 10 pages of results (ie, approximately 100 hits per keyword). Cookies and existing search histories were deleted before the start of each search, and the search results were downloaded into a Microsoft Excel file using a Chrome extension (ie, Export Search Results).

In addition, key intervention databases were searched by 2 reviewers (SvdK and SL). These included Beacon [37], Head to Health [38], and eMHPrac websites [39]. Finally, the reference lists and programs listed in the 2 existing scoping reviews on web-based depression [15] and anxiety [16] resources were searched by 2 reviewers (SvdK and SL) to identify any programs not included in the searches outlined. An overview of program developers and website links is provided in Multimedia Appendix 2.

### Selection of Web-Based Programs

**Figure 1:**
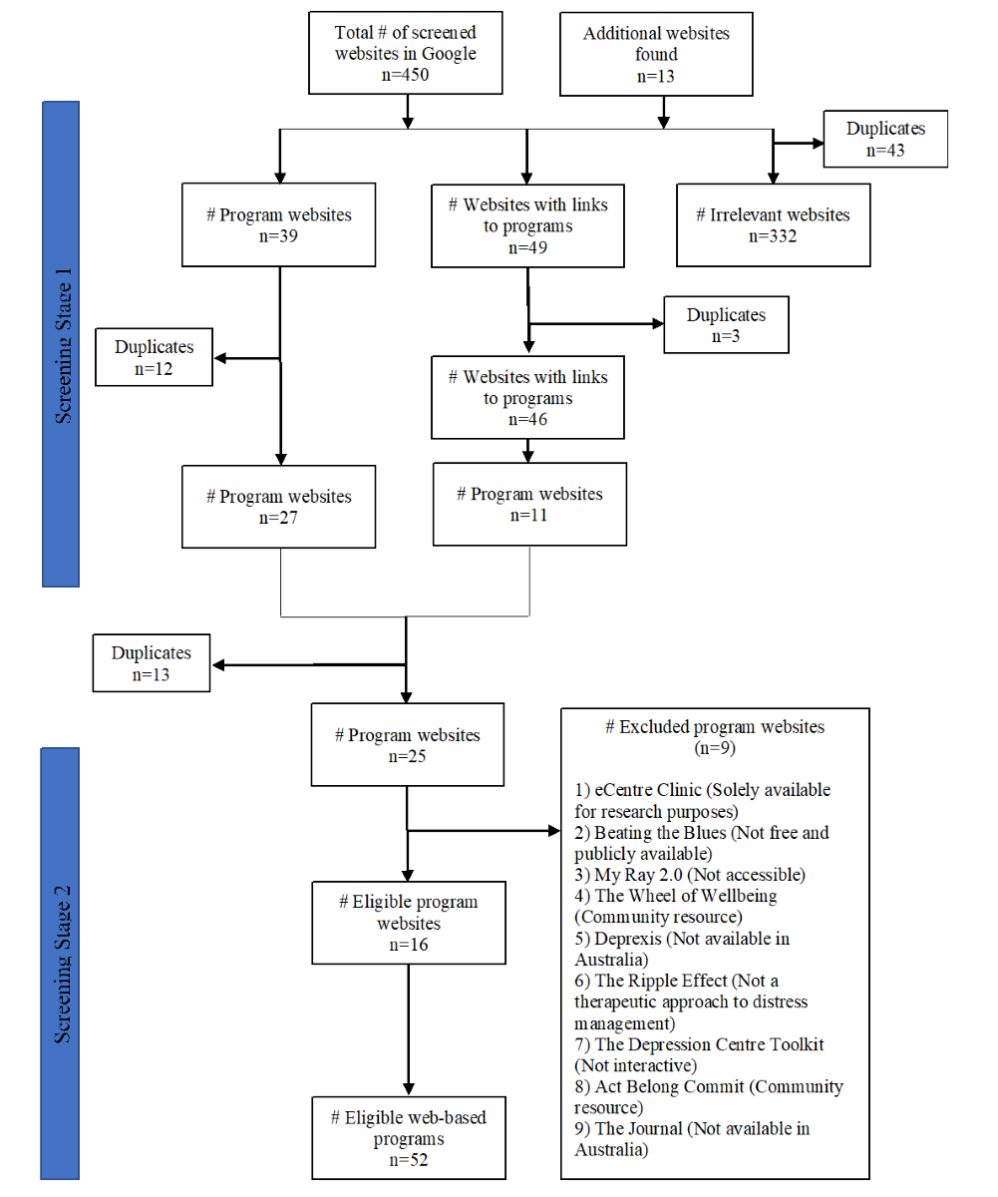
PRISMA (Preferred Reporting Items for Systematic Reviews and Meta-Analyses) flow diagram of program selection.

Screening of the search results to identify eligible web-based programs was completed in 2 stages. The first stage involved screening all search hyperlinks by 1 reviewer (SvdK) to eliminate websites that solely provided information on 1 of the outcomes or those that were clearly irrelevant (eg, mental health information only websites, YouTube videos, or blogs). The remainder of the websites was then divided into those that directly offered web-based programs and those that linked to web-based programs. Any duplicate websites were removed. The second stage involved screening potential eligible programs against the eligibility criteria by 2 reviewers (SvdK and SL; Figure 1).

### Data Extraction

A data extraction form was created to systematically evaluate each identified program. The extraction tool was adapted from a previously published framework designed to evaluate and report internet intervention studies [40]. This framework has been used in 2 recent scoping reviews examining web-based interventions for depression [15] and anxiety [16]. The main categories and subcategories of investigation were adapted from these studies (Table 1).

Websites were trialed and data extraction was undertaken by 3 reviewers (SvdK, SL, and GS) in November to December 2020, with the reviewers independently extracting data for each website so that the data extraction was performed in duplicate. Discrepancies in coding were resolved by discussion and, where necessary, investigated by the third reviewer. Where necessary, program authors were contacted to request access to the program. For 2 websites (MindSpot and Evolution Health), information was extracted from the public-facing pages where possible, and the remaining information was requested from the website authors or developers because of time and administrative constraints.

**Table 1:** Data extraction categories.

Main categories and subcategories		Evaluation focus
**Website characteristics**		
	Accessibility and credibility	How is the website accessed (free, via assessment, or via referral from a health care professional)? Registration requirement (if yes, what information is collected?) Mobile phone rendering (yes or no) Advertisements (if yes, relevant or irrelevant?)
	Authorship details	Presented contact details of authors or developers? (yes or no) Country of origin
**Program characteristics**		
	Focus and target population	Target issue Target audience
	Professional support	Therapist support (within the intervention, ability to link independent clinicians with the program)
	Other support	Peer support (did the program offer peer support, eg, forums? Was this monitored)?
	Program interactivity	This facet is addressed by type and dose of intervention
	Multimedia channel of delivery	Presentation format or mode of delivery (eg, how the content was delivered; text, audio, or video offered; use of character examples; and case scenarios)?
	Degree of synchronicity	Were email or SMS text message reminders or follow-up offered?
	Audience reach	This facet is addressed in focus and target population
**Intervention characteristics**		
	Model of change	Therapeutic approach
	Type and dose of intervention	Was the program structured or unstructured? Number of modules Suggested or set treatment length Self-assessments (were these mandatory or optional)?
	Intervention features	Worksheets (yes or no) Mood or symptom monitoring (yes or no) Diary (yes or no) Forums (yes or no) Other features (if yes, specify)
**Empirical evidence**		
	Program evaluation	Usability (did the program provide users with statistics on registered users, completion rates, attrition rates? Testimonial videos or case studies?) Empirical evidence (searched program website and Beacon^a^ database and contacted authors) Type of evidence (eg, RCT^b^, pre, or post?)
**Ethical issues**		
	Website	Privacy notice specified? Terms and conditions specified?
	Emergency	Does the program offer links to crisis or emergency contacts?

^a^Beacon is an Australian clinical web-based platform that describes different web-based self-help treatment programs.

^b^RCT: randomized controlled trial.

To identify relevant peer-reviewed papers and thereby explore the empirical evidence behind each program, we checked the program websites and the Beacon directory and contacted the program authors. A total of 3 reviewers (SvdK, DHB, and GS) then conducted a rapid review of the peer-reviewed evidence for each program. This was limited to evaluations of the complete web-based program rather than individual program elements or individual modules. In addition, the level of evidence was determined for each peer-reviewed study [41].

### Evaluation of Programs

A program evaluation scoring system was adapted from previously published guidelines for evaluating and reporting web-based intervention research [40], similar to Ashford et al [16]. The scale comprised 17 yes or no closed-ended questions (Textbox 1). Questions that were answered *yes* were given a score of 1 and questions that were answered *no* were scored 0. Questions that could not be evaluated were given a score of 0. Questions that were not relevant to the website were excluded from the final score (listed as “NA”). The final scores were calculated as a percentage of the number of relevant items for that website. A total of 3 reviewers (SvdK, SL, and GS) independently assessed the programs against the evaluation scoring system. Discrepancies were resolved through discussion.

**Program evaluation criteria (subcategories and questions).**

**Authorship details**

- Were the names and credentials of authors or organizations present?
- Were contact details provided?
- Was country of origin stated?

**Focus and target population**

- Was the primary focus, goals, or objectives of the intervention stated?
- Was the target audience or mental health issue defined?

**Professional support**

- Was there a statement of professional support (ie, clinicians involved in website development)?

**Multimedia channel of delivery**

- Did the program offer a multimedia content delivery (ie, a combination of text, video, graphics, and audio formats)?

**Model of change**

- Was the model of change (ie, type of therapy used) defined or stated?

**Type and dose of intervention**

- Were the number of modules or time to complete each module stated?
- Was the intervention tailored to the user or was it generic for all users (ie, was the program content individualized with username, characteristics, previous responses)?
- Was the program easy to navigate (eg, clear links to the home page and easily able to stop or start the program)?
- Was the information on what is covered in the intervention modules provided (ie, names of modules or a short description)?

**Program evaluation**

- Was evidence for the program provided to the user (ie, attrition data, success rate, completion rate, or number of users in the program or testimonials)?
- Has the program been empirically evaluated?

**Ethical issues**

- Was a unique username or password provided to users?
- Was a privacy notice specified?
- Were the terms and conditions specified?

### Synthesis of Results

The results were summarized quantitatively by main categories and subcategories, as displayed in Table 1. Where possible, descriptive statistics were used to summarize these findings.

## Results

### Selection of Web-Based Programs

As seen in Figure 1, 450 websites were screened through Google search, and an additional 13 websites were identified through database searches. Approximately 10.6% (49/463) of websites with links to potentially eligible programs and 39 potentially eligible programs were identified. Of the 49 websites, 25 (51%) program websites were screened for eligibility, of which 16 (64%) websites (leading to 52 programs) were included in the review. Of the 52 programs, 20 (39%) programs were classified as *web-based interactive*, and 32 (62%) programs were classified as *web-based with downloadable worksheets/resources*. These 2 types of programs were separated in the summary tables.

We only found 2 programs (BeyondNow and My Digital Health iConsider Life) designed to address distress in the context of suicidal ideation. It should be noted that the inclusion of BeyondNow was debated between team members, as we experienced difficulty in determining whether it fully fulfills the criteria of using an evidence-based therapeutic approach. Nonetheless, it is considered a valuable tool for the management of suicide ideation; therefore, it was included in this review.

### Website Characteristics

#### Accessibility and Credibility

Of the 52 programs, 36 (69%) were available at no cost and open to all Australians, 9 (17%) were accessible via referral from a health care professional (via This Way Up), 4 (8%) required an assessment before registration (via MindSpot), and 3 (6%) required sign-up to a research project (via My Digital Health; Tables 2 and 3). A free website (with 5/52, 10% programs) provided users with access to the basic course content without payment but required a paid upgrade to access additional features (Living Life to the Full). In total, 8% (4/52) of courses from Evolution Health were free to users. In addition, Evolution Health is a technology provider that licenses paid white label versions of its platform to organizations that contain only the course that the organization wants (eg, the smoking cessation course), thereby creating a *tailored* version of the platform for those organizations to distribute freely to their members.

Registration was required by 83% (43/52) of programs and most often required users’ names, emails, location (country, city, or postcode), and age. Approximately 6% (3/52) of programs created by My Digital Health were offered as part of a research study; of these, 2 programs, Life Flex and Life Flex–LGBQ (lesbian, gay, bisexual, and queer), required the completion of comprehensive demographic, mental health, and well-being questionnaires before access was granted. Approximately 17% (9/52) of programs did not require registration and allowed users anonymous access. All websites were accessible via mobile phones and did not contain advertisements, except for the 5 Living Life to the Full programs, which contained advertisements for e-books related to the course content.

**Table 2:** Program characteristics of web-based interactive programs.

Program		Focus and target population (if specified)	Access	Therapist support	Presentation format
**Depression**					
	eCouch—Depression Program	Depression, ≥16 years	Free	No	Text-based Graphics Characters or case scenarios Audio or video Comic slideshow format
	Mum2B—MoodBooster	Depression during pregnancy, expectant mothers	Free	No	Text-based Graphics Audio or video
	MumMood—Booster	Postnatal depression, women with postnatal depression	Free	Yes, optional	Text-based Graphics Audio or video
	OnTrack—Depression	Depression, ≥18 years	Free	No	Text-based Graphics Audio or video
**Anxiety**					
	eCouch—Anxiety and Worry Program	Anxiety and worry, ≥16 years	Free	No	Text-based Graphics Characters or case scenarios Audio or video Comic slideshow Format
	eCouch—Social Anxiety Program	Social anxiety, ≥16 years	Free	No	Text-based Graphics Characters or case scenarios Comic slideshow format
**Multi-issue**					
	MindSpot—Indigenous Wellbeing	Managing depression and anxiety, Aboriginal and Torres Strait Islanders, ≥18 years	Free following mandatory assessment and contact with MindSpot therapist	Yes, optional access to MindSpot therapist	Text-based Graphics Audio or video
	MindSpot—Mood Mechanic	Managing depression and anxiety, young adults, 18-25 years	Free following mandatory assessment and contact with MindSpot therapist	Yes, optional access to MindSpot therapist	Text-based Graphics Audio or video
	MindSpot—Wellbeing	Managing depression and anxiety, 26-65 years	Free following mandatory assessment and contact with MindSpot therapist	Yes, optional access to MindSpot therapist	Text-based Graphics Audio or video
	MindSpot—Wellbeing Plus	Managing depression and anxiety, ≥60 years	Free following mandatory assessment and contact with MindSpot therapist	Yes, optional access to MindSpot therapist	Text-based Graphics Audio or video
	MoodGym	Anxiety and depression	Free	No	Text-based Graphics Audio or video
	MyCompass	Mild to moderate depression, anxiety and stress, ≥18 years	Free	No	Text-based Graphics Audio or video
	My Digital Health—Life Flex	Anxiety or depression, ≥18 years	Free (via research study participation)	Yes, ability to connect independent clinician to program	Text-based Graphics Audio or video
	My Digital Health—Life Flex LGBQ^a^	Anxiety or depression, ≥18 years who are part of the LGBQ community	Free (via research study participation)	Yes, ability to connect independent clinician to program	Text-based Graphics Audio or video
**General well-being**					
	eCouch—Bereavement and Loss Program	Bereavement and loss, ≥16 years	Free	No	Text-based Graphics Characters or case scenarios Audio or video Comic slideshow format
	eCouch—Divorce and separation	Divorce and separation, ≥16 years	Free	No	Text-based Graphics Characters or case scenarios Comic slideshow format
	ifarmwell	Well-being, farmers, ≥18 years	Free	No	Text-based Graphics Audio or video
	The Desk	Well-being, Australian tertiary students	Free	No	Text-based Graphics Audio or video
**Suicidal ideation**					
	My Digital Health—iConsiderLife	Decision support crisis digital health program, ≥18 years	Free (via research study participation)	No	Text-based Audio or video
	BeyondNow	Suicide safety planning	Free	N/A^b^	Text-based Workbook or planner

^a^LGBQ: lesbian, gay, bisexual, and queer.

^b^N/A: not applicable.

**Table 3:** Program characteristics of web-based programs with downloadable worksheets or resources.

Program			Focus and target population (if specified)		Access		Therapist support		Presentation format
**Depression**									
	CCI^a^—Depression	Depression		Free		No		Text workbook with images	
	Evolution Health—Overcoming Depression	Depression, ≥16 years		Free		Not in free version		Text Gamified quiz	
	Mental health Online—Depression Online Program	Depression, ≥18 years		Free		Yes, optional access to e-therapists		Text-based Graphics Audio or video Characters or case scenarios	
	This Way Up—The Depression Course	Depression, ≥18 years		Free when referred by clinician		Yes, independent clinician allowed access		Text-based Graphics Audio or video Characters or case scenarios Comic slideshow format	
	Students Against Depression	Depression, students		Free		No		Text-based	
**Anxiety**									
	CCI—Health and Anxiety	Health anxiety		Free		No		Text workbook with images	
	CCI—Panic	Panic attacks		Free		No		Text workbook with images	
	CCI—Social Anxiety	Social anxiety		Free		No		Text workbook with images	
	CCI—Worry and Rumination	Worry		Free		No		Text workbook with images	
	Evolution Health—Overcoming Anxiety	Anxiety, ≥16 years		Free		Not in free version		Text Gamified quiz	
	Evolution Health—Managing Anxiety Course	Anxiety, ≥16 years		Free		Not in free version		Text Gamified quiz	
	Mental Health Online—GAD^b^ program	GAD, ≥18 years		Free		Yes, optional access to e-therapists		Text-based Graphics Audio or video Characters or case scenarios	
	Mental Health Online—Panic STOP!	Panic attacks, ≥18 years		Free		Yes, optional access to e-therapists		Text-based Graphics Audio or video Characters or case scenarios	
	Mental Health Online—Social Anxiety Online Program	Social anxiety, ≥18 years		Free		Yes, optional access to e-therapists		Text-based Graphics Audio or video Characters or case scenarios	
	This Way Up—Health Anxiety Course	Worry about health, ≥18 years		Free when referred by clinician		Yes, independent clinician allowed access		Text-based Graphics Audio or video Characters or case scenarios Comic slideshow format	
	This Way Up—Panic Attacks Course	Panic attacks, ≥18 years		Free when referred by clinician		Yes, independent clinician allowed access		Text-based Graphics Audio or video Characters or case scenarios Comic slideshow format	
	This Way Up—Social Anxiety Course	Social anxiety, ≥18 years		Free when referred by clinician		Yes, independent clinician allowed access		Text-based Graphics Audio or video Characters or case scenarios Comic slideshow format	
	This Way Up—Worry Course (GAD)	GAD, ≥18 years		Free when referred by clinician		Yes, independent clinician allowed access		Text-based Graphics Characters or case scenarios Comic slideshow format	
**Multi-issue**									
	Mental Health Online—Made-4-Me Program	Can pick up to 3 areas: depression, GAD, panic disorder, PTSD^c^, social anxiety, OCD^d^, ≥18 years		Free		Yes, optional access to e-therapists		Text-based Graphics Audio or video Characters or case scenarios	
	This Way Up—Mindfulness-Based CBT^e^ Course	Depression and anxiety, ≥18 years		Free when referred by clinician		Yes, independent clinician allowed access		Text-based Graphics Audio or video Characters or case scenarios Comic slideshow format	
	This Way Up—Mixed Depression and Anxiety Course	Depression and anxiety, ≥18 years		Free when referred by clinician		Yes, independent clinician allowed access		Text-based Graphics Audio or video Characters or case scenarios Comic slideshow format	
	This Way Up—MUMentum Pregnancy	Anxiety and low mood, adults <36 week pregnant		Free when referred by clinician		Yes, independent clinician allowed access		Text-based Graphics Characters or case scenarios Comic slideshow format	
	This Way Up—MUMentum Postnatal	Anxiety and low mood, adults >36 weeks pregnant or have given birth within the last 12 months		Free when referred by clinician		Yes, independent clinician allowed access		Text-based Graphics Characters or case scenarios Comic slideshow format	
**General** **well-being**									
	CCI—Tolerating Distress	Distress		Free		No		Text workbook with images	
	Evolution Health—Grief and Loss	Grief, ≥16 years		Free		Not in free version		Text Gamified quiz	
	Living Life to the Full for Adults	Emotional well-being, adults		Free for the basic course; can upgrade for a fee		No		Text-based Graphics Audio or video Comic slideshow format	
	Living Life to the Full for Farming Communities	Emotional well-being, farming communities		Free for the basic course; can upgrade for a fee		No		Text-based Graphics Audio or video Comic slideshow format	
	Living Life to the Full with God	Emotional well-being, people of faith		Free for the basic course; can upgrade for a fee		No		Text-based Graphics Audio or video Comic slideshow format	

^a^CCI: Center for Clinical Interventions.

^b^GAD: generalized anxiety disorder.

^c^PTSD: posttraumatic stress disorder.

^d^OCD: obsessive compulsive disorder.

^e^CBT: cognitive behavioral therapy.

#### Authorship Details

The review was limited to programs that were *accessible* to Australians. Unsurprisingly, most of these programs identified Australia as their country of origin (40/52, 77%), although other countries of origin were Scotland (5/52, 10%), Canada (4/52, 8%), the United States (in collaboration with Australia; 2/52, 4%) and the United Kingdom (1/52, 2%). Contact details for the authors or developers were provided by almost all websites (51/52, 98%). An overview of program developers and website links is provided in Multimedia Appendix 2.

### Program Characteristics

Table 2 provides an overview of program characteristics for web-based interactive programs and Table 3 for web-based programs with downloadable worksheets or resources.

#### Focus and Target Population

Overall, 17% (9/52) of programs specifically addressed depression, 29% (15/52) of programs addressed anxiety, 25% (13/52) of programs addressed multiple issues, 25% (13/52) of programs addressed general well-being, and 4% (2/52) of programs addressed suicidal ideation. Most programs specifically stated that their target audience was the general adult population (aged ≥16 years or ≥18 years; 26/52, 50%), with other (9/52, 17%) programs not specifying a target group but appearing to be designed for adults, based on content. Several programs were designed for specific target populations, including expectant mothers or new parents (6/52, 12%), tertiary students (3/52, 6%), farmers or rural communities (2/52, 4%), church-going adults (1/52, 2%), the LGBQ community (1/52, 2%), Aboriginal and Torres Strait Islander people (1/52, 2%), young adults (1/52, 2%), adults aged 26 to 65 years (1/52, 2%), and older adults (1/52, 2%).

#### Support and Features

Approximately 88% (46/52) of programs provided a statement of professional support, that is, mental health clinicians or researchers were involved in the programs’ development. Of the 52 programs, 11 (21%) programs allowed users to invite their independent clinician to monitor their progress in the course, and a further 10 (19%) programs offered optional therapist support while engaging in the program. Approximately 8% (4/52) of programs (Evolution Health) did not offer therapist support through the free version of their programs; however, therapist support was an optional inclusion for paid versions of the program licensed to organizations. Only 8% (4/52) of programs (Evolution Health) enabled peer support, such as through forums, and in all cases, this was expert-moderated. Most programs offered multiple forms of information delivery, with text, graphics, audio or video, characters, and case scenarios as common features. Email or SMS text message reminders were available 60% (31/52) of the programs.

### Intervention Characteristics

#### Overview

The intervention characteristics of web-based interactive programs and web-based programs with downloadable worksheets or resources are shown in Tables 4 and 5, respectively.

**Table 4:** Intervention characteristics of web-based interactive programs.

Program		Therapeutic approach	Program structure	Modules (length)	Intervention features
**Depression**					
	eCouch—Depression Program	CBT^a^+IPT^b^	Structured	3 sections	Worksheets Mood or symptom monitoring Diary
	Mum2B—MoodBooster	CBT	Structured	6 modules (6 weeks)	Worksheets Mood or symptom monitoring
	MumMood—Booster	CBT	Structured	6 modules (6 weeks)	Worksheets Mood or symptom monitoring
	OnTrack—Depression	CBT	Structured	6 modules (8 weeks)	Worksheets Mood or symptom monitoring Diary Reminder or calendar feature
**Anxiety**					
	eCouch—Anxiety and Worry Program	CBT+IPT	Structured	3 sections	Worksheets Mood or symptom monitoring Diary
	eCouch—Social Anxiety Program	CBT+IPT	Unstructured	6 sections	Worksheets
**Multi-issue**					
	MindSpot—Indigenous Wellbeing	CBT	Structured	5 modules (8 weeks)	Worksheets
	MindSpot—Mood Mechanic	CBT	Structured	5 modules (8 weeks)	Worksheets
	MindSpot—Wellbeing	CBT	Structured	5 modules (8 weeks)	Worksheets
	MindSpot—Wellbeing Plus	CBT	Structured	5 modules (8 weeks)	Worksheets
	MoodGym	CBT	Structured	5 modules	Worksheets Mood or symptom monitoring
	MyCompass	CBT+ IPT+positive psychology	Unstructured	14 activities (recommend 7 weeks)	Worksheets Mood or symptom monitoring Diary
	My Digital Health—Life Flex	Biopsychosocial approach+CBT+positive psychology	Structured (optional or mandatory depending on RCT^c^ arm)	7 modules (scheduled release over 7 weeks in 1 RCT arm)	Worksheets Mood or symptom monitoring Diary Option to connect Fitbit or other health monitoring tools Safety planning tool
	My Digital Health—Life Flex LGBQ^d^	Biopsychosocial approach+CBT+positive psychology	Structured (optional or mandatory depending on RCT arm)	7 modules (scheduled release over 7 weeks in 1 RCT arm)	Worksheets Mood or symptom monitoring Diary Option to connect Fitbit or other health monitoring tools Safety planning tool
**General well-being**					
	eCouch—Bereavement and Loss Program	CBT	Structured	2 sections	Worksheets Mood or symptom monitoring Diary
	eCouch—Divorce and Separation	CBT	Structured	3 sections	Worksheets Mood or symptom monitoring Diary
	ifarmwell	ACT^e^	Structured	5 modules (10 weeks)	Worksheets Personalized script
	The Desk	CBT+mindfulness+positive psychology	Unstructured	4 modules	Mood or symptom monitoring Reminder or calendar feature
**Suicidal ideation**					
	My Digital Health—iConsiderLife	No	Structured	6 pathways	Worksheets
	BeyondNow	N/A^f^	Unstructured	No modules	Process of completing plan is like a worksheet

^a^CBT: cognitive behavioral therapy.

^b^IPT: interpersonal psychotherapy.

^c^RCT: randomized controlled trial.

^d^LGBQ: lesbian, gay, bisexual, and queer.

^e^ACT: acceptance and commitment therapy.

^f^N/A: not applicable.

**Table 5:** Intervention characteristics of web-based programs with downloadable worksheets or resources.

Program			Therapeutic approach		Program structure		Modules (length)		Intervention features
**Depression**									
	CCI^a^—Depression	CBT^b^		Structured, optional		9 modules		Worksheets Mood or symptom monitoring Diary	
	Evolution Health—Overcoming Depression	CBT+motivational interviewing		Structured but not mandatory to follow structure		9 modules		Worksheets Experiments to try Symptom tracker diary Optional self-assessments Member forum Messaging with others Goal setting	
	Mental Health Online—Depression Online Program	CBT		Structured, optional		11 modules (recommend 12 weeks)		Worksheets Mood or symptom monitoring	
	This Way Up—The Depression Course	CBT		Structured		6 modules (3 months)		Worksheets Mood or symptom monitoring Reminder or calendar feature	
	Students Against Depression	CBT		Structured, optional		6 modules (recommend 6 weeks)		Worksheets Diary Safety planning tool	
**Anxiety**									
	CCI—Health and Anxiety	CBT		Structured, optional		9 modules		Worksheets Diary	
	CCI—Panic	CBT		Structured, optional		12 modules		Worksheets Mood or symptom monitoring Diary	
	CCI—Social Anxiety	CBT		Structured, optional		10 modules		Worksheets Mood or symptom monitoring Diary	
	CCI—Worry and Rumination	Metacognitive therapy		Structured, optional		10 modules		Worksheets Mood or symptom monitoring Diary	
	Evolution Health—Overcoming Anxiety	CBT+motivational interviewing		Structured but not mandatory to follow structure		9 modules		Worksheets Experiments to try Mood tracker diary Optional self-assessments Member forum Messaging with others Goal setting	
	Evolution Health—Managing Anxiety Course	CBT+motivational interviewing		Structured but not mandatory to follow structure		1 module		Worksheets Experiments to try Optional self-assessments Member forum Messaging with others Goal setting	
	Mental Health Online—GAD^c^ Program	CBT		Structured, optional		12 modules (recommend 12 weeks)		Worksheets Mood or symptom monitoring	
	Mental Health Online—Panic STOP!	CBT		Structured, optional		12 modules (recommend 12 weeks)		Worksheets Mood or symptom monitoring	
	Mental Health Online—Social Anxiety Online Program	CBT		Structured, optional		12 modules (recommend 12 weeks)		Worksheets Mood or symptom monitoring	
	This Way Up—Health Anxiety Course	CBT		Structured		6 modules (3 months)		Worksheets Mood or symptom monitoring Reminder or calendar feature	
	This Way Up—Panic Attacks Course	CBT		Structured		6 modules (3 months)		Worksheets Mood or symptom monitoring Reminder or calendar feature	
	This Way Up—Social Anxiety Course	CBT		Structured		6 modules (3 months)		Worksheets Mood or symptom monitoring Reminder or calendar feature	
	This Way Up—Worry Course (GAD)	CBT		Structured		6 modules (3 months)		Worksheets Mood or symptom monitoring Reminder or calendar feature	
**Multi-issue**									
	Mental Health Online—Made-4-Me Program	CBT		Structured, optional		11 modules (recommend 12 weeks)		Worksheets Mood or symptom monitoring	
	This Way Up—Mindfulness-based CBT Course	CBT+mindfulness		Structured		6 modules (3 months)		Worksheets Mood or symptom monitoring Reminder or calendar feature	
	This Way Up—Mixed Depression and Anxiety Course	CBT		Structured		6 modules (3 months)		Worksheets Mood or symptom monitoring Reminder or calendar feature	
	This Way Up—MUMentum Pregnancy	CBT		Structured		3 modules (4-6 weeks)		Worksheets Mood or symptom monitoring Reminder or calendar feature	
	This Way Up—MUMentum Postnatal	CBT		Structured		3 modules (4-6 weeks)		Worksheets Mood or symptom monitoring Reminder or calendar feature	
**General well-being**									
	CCI—Tolerating Distress	DBT^d^		Structured, optional		4 modules		Worksheets Mood or symptom monitoring Diary	
	Evolution Health—Grief and Loss	CBT+motivational interviewing		Structured but not mandatory to follow structure		1 module		Worksheets Experiments to try Optional self-assessments Member forum Messaging with others Goal setting	
	Living Life to the Full for Adults	CBT		Structured, optional		8 modules (recommend 8 weeks)		Worksheets Mood or symptom monitoring Blog Reminder or calendar feature	
	Living Life to the Full for Farming Communities	CBT		Structured, optional		5 modules		Worksheets Mood or symptom monitoring Diary Blog Reminder or calendar feature	
	Living Life to the Full with God	CBT		Structured, optional		8 modules (recommend 8 weeks)		Worksheets Mood or symptom monitoring Diary Blog Reminder or calendar feature	
	Living Life to the Full —Enjoy Your Baby	CBT		Structured, optional		5 modules		Worksheets Blog Reminder or calendar feature	
	Living Life to the Full —Enjoy Your Bump	CBT		Structured, optional		5 modules		Worksheets Mood or symptom monitoring Blog Reminder or calendar feature	
	This Way Up—Coping with Stress Course	CBT		Structured		4 modules (3 months)		Worksheets Mood or symptom monitoring Reminder feature Calendar feature	
	This Way Up—The Student Wellbeing Course	CBT		Structured		8 modules (3 months)		Worksheets Mood or symptom monitoring Reminder or calendar feature	

^a^CCI: Centre for Clinical Interventions.

^b^CBT: cognitive behavioral therapy.

^c^GAD: generalized anxiety disorder.

^d^DBT: dialectical behavior therapy.

#### Therapeutic Approach or Model of Change

On the basis of either self-descriptions or the clinical judgment of the authors, 90% (47/52) of programs were based on cognitive behavioral therapy (CBT), either with 21% (11/52) or without 69% (36/52) other therapeutic approaches. In combination with CBT, some programs included motivational interviewing (4/52, 8%), interpersonal therapy (4/52, 8%), or mindfulness (1/52, 2%). Only 2% (1/52) of programs (ifarmwell) was based on acceptance and commitment therapy (ACT), 2% (1/52) of programs (Centre for Clinical Interventions [CCI]: Tolerating Distress) on dialectical behavior therapy (DBT), and 2% (1/52) of programs (BeyondNow) on problem solving (ie, how to keep self safe or safety planning).

#### Type and Dose of Intervention

In total, 48% (25/52) of programs were structured or *tunneled*, where participants were led through a series of modules or sessions in a specific order. A further 42% (22/52) of programs recommended to follow the program in a specific order but allowed users to choose the order in which they completed them. Approximately 4% (2/52) of programs were part of an RCT research project that had open access and allocated participants to 1 of 2 *therapy structure* conditions: mandatory (participants did not choose the order) or optional (participants were given the freedom to choose the order of completion). Approximately 6% (3/52) of programs were unstructured.

The number of modules or activities offered within a program ranged from 1 to 14 (mean 6.51, SD 3.03), except for 1 program (BeyondNow), which offered a safety planner instead of modules. The recommended time to complete the modules ranged from 1 week to 3 months (specified for 34/52, 65% of programs). Approximately 33% (17/52) of programs did not recommend a set treatment length. Self-assessments were included in 79% (41/52) programs, and it was mandatory in 56% (29/52) of programs and optional in 23% (12/52) of programs.

#### Intervention Features

All programs, except 4% (2/52; The Desk and iConsider Life), featured worksheets, either within the web-based intervention or as downloadable forms to be completed offline. Diaries were featured in 46% (24/52) of programs, and 75% (39/52) of programs included some type of mood or symptom monitoring.

### Empirical Evidence: Program Evaluation

Out of 17 possible evaluation items, 75% (39/52) of programs could be evaluated against all 17 items, 15% (8/52) against 94% (16/17) of items, and 10% (5/52) against 76% (13/17) of items (Table 6). In total, scores ranged from 44% to 100%, with an average score of 85% (SD 10.7%). More specifically, of the 75% (39/52) of programs that provided values for all 17 items, scores varied between 65% and 94%, with an average score of 87% (SD 7%). This was between 44% and 81%, with an average of 69% (SD 11%), for programs that provided values for 16 items, and between 85% and 100%, with an average of 95% (SD 7%), for programs that provided values for 13 items. Individual answers to the program evaluation criteria are shown in Multimedia Appendix 3.

**Table 6:** Type of research conducted to evaluate eligible programs, including the level of evidence and process evaluation scores (N=52).

Program	Type of research evaluation studies	Level of evidence^a^	Total number of relevant evaluation items, N	Process evaluation score, n (%)^b,c^
BeyondNow	Feasibility and effectiveness study [42]	Level III-3	13	11 (85)
CCI^d^—Depression	Website: not specified Beacon^e^: not reviewed	—^f^	16	11 (69)
CCI—Health Anxiety	Preliminary pre–post study [43]	Level IV	16	12 (75)
CCI—Panic	Website: not specified Beacon: not reviewed	—	16	11 (69)
CCI—Social Anxiety Course	Website: not specified Beacon: not reviewed	—	16	11 (69)
CCI—Tolerating Distress	Website: not specified Beacon: not reviewed	—	16	11 (69)
CCI—Worry and Rumination	Preliminary pre–post study [43]	Level IV	16	12 (75)
eCouch—Anxiety and Worry	Adapted from MoodGym RCT^g^ [44,45]	Level II	17	13 (76)
eCouch—Bereavement and Loss	Adapted from MoodGym	—	17	11 (65)
eCouch—Depression	Adapted from MoodGymRCT [46-50]	Level II	17	13 (76)
eCouch—Divorce and Separation	Adapted from MoodGym	—	17	12 (71)
eCouch—Social Anxiety	Adapted from MoodGym 2-group RCT and to assess effectiveness and cost-effectiveness [12] Pre–post with control group and randomized [51]	Level II Level II	17	14 (82)
Evolution Health—Overcoming Anxiety	Pre–post longitudinal study [52] Pilot RCT [53] User characteristics [54]	Level IV Level IV Level IV	17	15 (88)
Evolution Health—Overcoming Depression	Website: not specified Beacon: not reviewed	—	17	14 (82)
Evolution Health—Grief and Loss	Website: not specified Beacon: not reviewed	—	17	14 (82)
Evolution Health—Managing Anxiety	Pre–post longitudinal study [52]	Level IV	17	15 (88)
ifarmwell	Website: currently being evaluated Beacon: not reviewed	—	17	15 (88)
Living Life to the Full for Adults	Comparative clinical feasibility study [55] Feasibility [56]	Level III-3 Level III-3	17	16 (94)
Living Life to the Full for Farming Communities	Website: not specified Beacon: not reviewed	—	17	15 (88)
Living Life to the Full with God	Website: not specified Beacon: not reviewed	—	17	15 (88)
Living Life to the Full—Enjoy Your Baby	Website: not specified Beacon: not reviewed	—	17	15 (88)
Living Life to the Full—Enjoy Your Bump	Website: not specified Beacon: not reviewed	—	17	15 (88)
Mental Health Online—Depression Online	Uncontrolled pre–post treatment study [57,58]	Level IV	17	15 (88)
Mental Health Online—Generalized Anxiety Disorder	Uncontrolled pre–post treatment study [57] Pre–post treatment [59] Pre- to posttreatment quasi-experimental (participant choice) [60]	Level IV Level IV Level IV	17	15 (88)
Mental Health Online—Made 4 Me	Website: not specified Beacon: not reviewed	—	17	15 (88)
Mental Health Online—Panic STOP!	Uncontrolled pre–post treatment study [57] Pre–post treatment [59] Participant choice quasi-experimental trial [60]	Level IV Level IV Level IV	17	15 (88)
Mental Health Online—Social Anxiety Online	Pre–post treatment study [59] Participant choice quasi-experimental trial [60]	Level IV Level IV	17	15 (88)
MindSpot—Indigenous Wellbeing Course	Prospective uncontrolled observational cohort study [61]	Level IV	13	13 (100)
MindSpot—Mood Mechanic Course	Single-arm, open trial [62] RCT [63]	Level III-3 Level II	13	13 (100)
MindSpot—Wellbeing	Cost-effectiveness [64] Feasibility trial [65] RCT [66-73] 12-month follow-up RCT [62] Single group open trial [74]	Level III-3 Level II Level II Level III-3	13	13 (100)
MindSpot—Wellbeing Plus	Cost-effectiveness [75,76] Feasibility study [75,77] Implementation [78] RCT [75,76,79,80]	Level II Level II and III-3 Level II Level II	13	13 (100)
MoodGym	Acceptability study [81] Implementation [82] Follow-up outcome analysis [83] Program use [84] RCTs [10,17,81,82,85-99] School or class trials [100,101] Compliance [102]	Level II Level II Level II Level II Level III-2 Level II	17	15 (88)
Mum2BMoodBooster	Website: currently being evaluated Beacon: not reviewed	—	17	14 (82)
MumMoodBooster	Acceptability study [103,104] Feasibility study [104] RCT [105]	Level III-3 Level III-3 Level II	17	15 (88)
myCompass	Feasibility study [106] Open trial [107] RCT [108-110]	Level II-3 Level III-3 Level II	17	16 (94)
My Digital Health—iConsiderLife	Website: currently being evaluated Beacon: not reviewed	—	16	13 (81)
My Digital Health—Life Flex	Website: currently being evaluated Beacon: not reviewed	—	17	15 (88)
My Digital Health—Life Flex LGBQ^h^	Website: currently being evaluated Beacon: not reviewed	—	17	15 (88)
OnTrack—Depression	Website: not specified Beacon: not reviewed	—	17	13 (76)
The Desk	Website: not specified Beacon: not reviewed	—	17	14 (82)
This Way Up—Coping with Stress Course	Website: not specified Beacon: not reviewed	—	17	15 (88)
This Way Up—The Depression Course	Nonrandomized comparison study [111] Open trial [112-117] RCT [111,118-124] Adherence [125]	Level III-2 Level III-3 Level II-3 Level III-3	17	16 (94)
This Way Up—Health Anxiety Course	Open trial [126,127] RCT [128]	Level III-3 Level II	17	16 (94)
This Way UpMindfulness-based CBT^i^ Course	Open trial [129] RCT [130]	Level III-3 Level II	17	16 (94)
This Way Up—Mixed Depression and Anxiety Course	Open trial [74,111,131-133] RCT [72,131,134]	Level III-3 Level II	17	16 (94)
This Way Up—MUMentum Pregnancy	RCT [135]	Level II	17	16 (94)
This Way Up—MUMentum Postnatal	RCT [136]	Level II	17	16 (94)
This Way Up—Panic Attacks Course	Open trial [137] RCT [71,72,138-140]	Level III-3 Level II	17	16 (94)
This Way Up—Social Anxiety Course	Open trial [141] RCT [71,72,138,142-146]	Level III-3 Level II	17	16 (94)
This Way Up—Student Wellbeing Course	Website: not specified Beacon: not reviewed	—	17	15 (88)
This Way Up—Worry Course (GAD^j^)	Nonrandomized comparison study [111] Open trial [112,133,147,148] RCT [71,72,138,149-151]	Level III-2 Level III-3 Level II	17	16 (94)
Students Against Depression	Website: currently being evaluated Beacon: not reviewed	—	16	7 (44)

^a^Level of evidence determined from the National Health and Medical Research Council evidence hierarchy by intervention studies [41]; level I: systematic review; level II: randomized controlled trials; level III-1: pseudorandomized controlled trial; level III-2: comparative study with controls; level III-3: comparative study without controls; and level IV: case series.

^b^A program evaluation scoring system was adapted from previously published guidelines for evaluating and reporting web-based intervention research [40].

^c^Owing to access restrictions, not all programs could be evaluated against all 17 items, which may have resulted in higher scores.

^d^CCI: Centre for Clinical Interventions.

^e^Beacon is an Australian clinical web-based platform that describes different web-based self-help treatment programs.

^f^Evaluation studies are not available.

^g^RCT: randomized controlled trial.

^h^LGBQ: lesbian, gay, bisexual, and queer.

^i^CBT: cognitive behavioral therapy.

^j^GAD: generalized anxiety disorder.

Evidence of the program’s usability (ie, attrition data, success rate, completion rate, or number of users in the program or testimonials) was provided for 52% (27/52) of programs. In many cases, where a website offered ≥1 program, these results were provided for the suite of programs listed on the website generally rather than for the specific programs (eg, Evolution Health and This Way Up). Approximately 56% (29/52) of programs were supported by some type of evaluation; 59% (17/29) had undergone effectiveness trials, and 41% (12/29) had empirical evidence of both effectiveness and acceptability (ie, level III and level IV evidence). Approximately 68% (19/29) of the evaluated programs had undergone evaluation via RCT (ie, level II evidence). Of the 23 programs without evaluation data, 6 (26%) are currently in the process of being evaluated. All 52 programs and the type of evaluation studies conducted, including the level of evidence, are shown in Table 6 [41]. A rapid review of the evidence for each of these programs’ evaluations is provided in Multimedia Appendix 4 [10,12,17,42-151].

### Legal and Ethical Issues

The vast majority of programs provided privacy notices (50/52, 96%) and terms and conditions (51/52, 98%). Approximately 88% (46/52) of programs provided crisis links in the form of telephone numbers for emergency services, numbers of helplines or links to websites, and other resources. Only 1 website did not offer crisis links for its programs (CCI); this website was developed by the Western Australian Department of Health to provide evidence-based web-based resources to support practitioners, as well as to provide self-help materials for individuals. The terms and conditions of the website state that the information is provided for information purposes only.

## Discussion

### Principal Findings

The purpose of this scoping review was to summarize the freely available web-based resources based on an evidence-based therapeutic approach for Australian adults seeking self-help for depression, anxiety, suicidal ideation, or general well-being. We sought to describe their characteristics, including therapeutic approaches, key features, and the quality of evidence behind them and thereby produce an accessible summary to inform clinicians’ selection of web-based interventions for their patients. This review builds on past reviews of web-based resources for depression [15] and anxiety [16]. To our knowledge, this is the first review of publicly available interventions that includes both suicidal ideation and general well-being.

A total of 52 web-based programs were identified, of which 20 (39%) were classified as web-based interactive programs, and 32 (62%) were classified as web-based programs with downloadable worksheets or resources. Of these 52 programs, 29 (56%) had been empirically evaluated, and most of the evidence was assessed as level II or III studies. This is similar to past reviews, which found that 50% of the programs addressing anxiety had undergone empirical evaluation [16], and 38% of programs addressing depression had been evaluated via an RCT (ie, level II studies) [15]. A total of 6 additional programs in this review were also currently undergoing evaluation at the time of writing (ifarmwell, My Digital Health programs, Mum2BMoodBooster, and Students Against Depression).

The evaluation of the included programs showed that, on average, programs scored 85%, indicating that the included web-based programs are generally of good quality. The lowest score was for Students Against Depression (44%) as it did not include information about the developers or the evidence behind the program, privacy notices, terms and conditions. However, Students Against Depression was one of the programs that offered downloadable modules, similar to the CCI programs, and it is currently undergoing evaluation, which may lead to a higher evaluation score once completed. The CCI programs, which scored third to last but are highly regarded by clinicians, included a privacy notice and terms and conditions, and their scores ranged between 69% and 75%. The highest scores were given to the 4 programs by MindSpot, which could only be evaluated against 77% (13/17) of the items. These programs could not be evaluated on more of the items because of restrictions on accessing the program, which may have resulted in higher scores.

Almost all programs reported that they were based on CBT, either with or without other therapeutic approaches. Only 1 program, ifarmwell, was based on ACT. Although traditional CBT focuses on challenging unhelpful thoughts, ACT focuses on defusing from and accepting them and finding value-consistent ways of living despite the circumstances to build *psychological flexibility* [152]. A recent international systematic review and meta-analysis of internet-based ACT programs between 2009 and 2019 [153] found 25 efficacy studies in a variety of clinical and nonclinical populations. Overall, the pooled results showed a reduction in symptoms of depression and anxiety and an improvement in quality of life and psychological flexibility at postassessment, with results maintained at follow-up assessments. Given this evidence, it is surprising that not more ACT-based web- interventions are available in Australia. However, that review focused on research-driven studies and not publicly available programs; therefore, none of the studies examined by Thompson et al [153] overlapped with this review. Similarly, only 1 program was based on DBT, which differs from CBT in that its main focus is on helping people change their behavior patterns instead of changing dysfunctional beliefs [154,155]. It also focuses on distress tolerance, which is more like ACT than CBT, and includes mindfulness and interpersonal skills training. A review of clinical effectiveness and guidelines showed that DBT is not statistically significantly greater than comparators in reducing depressive and anxiety symptoms [156].

The results of the current review found that 19% (10/52) of programs provided free therapist support, and 21% (11/52) allowed users to link in their existing clinicians. Research examining whether therapist-guided versus self-guided web-based interventions are more, less, or equally effective has produced mixed results. A recent meta-analysis of 21 studies of web-based programs targeting depression found no significant differences in the effectiveness of the programs to prevent depression based on whether they were guided or not [31]. In contrast, another meta-analysis of studies reported greater effect sizes for outcomes from interventions for depression and anxiety that were guided compared with effect sizes for those that were not [153]. However, these differences may be because of the type of therapy given, as the former was based on CBT and the latter on ACT. In addition, the use of email or SMS text message reminders or messages of encouragement may compensate for the lack of guidance in some programs. Guided interventions are not always practical to implement and may be expensive and hard to sustain and limit the accessibility of the program. Therefore, future research should continue to examine the impact of incorporating, or not incorporating, these program components and alternatives.

This review found that roughly half of the programs were structured or tunneled; a further 22 programs provided a recommended structure (but did not require that the user follow this), and 3 programs were unstructured, allowing the user choice in how and when they completed different modules. Research evidence is inconclusive on the manner in which web-based materials are best organized [157]. A recent systematic review found inconclusive evidence linking website structure and behavioral or health outcomes [158]. They reported that the number of peer-reviewed studies that manipulated website structure to examine its effects on outcome measures was too few to enable conclusive comments to be made. Other studies have provided evidence that allowing unstructured use of websites may increase engagement with the website; however, this may or may not translate into behavior change [159]. Therefore, more empirical evidence is required.

Most resources were designed for adults generally, although a few included programs were designed for specific populations such as farmers, tertiary students, and expectant or new mothers. There were no programs identified in our searches that were designed for hard-to-reach groups such as culturally and linguistically diverse populations or men, and there was only 1 for people from an Aboriginal or Torres Strait Islander cultural background. Web-based interventions provide a useful avenue for administering targeted mental health support to populations who may face barriers to accessing traditional services for cultural reasons. These results highlight the need for more research and development of programs that meet the needs of these at-risk and underserved groups.

### Limitations

The use of a scoring system to evaluate and compare programs was based on a framework for evaluating internet-based interventions [40], as adapted in subsequent research [15,16]. This is a useful tool for comparisons across programs and between this research and past studies. However, items may not reflect the overall true value of a program to a particular consumer, with many elements of the program not being included in this scale and not necessarily being quantifiable as a yes or no item. Future research could extend this by examining how consumers and practitioners select an appropriate resource and the factors that they consider to be important in this choice. In addition, although we evaluated the usability of the programs, no completion rates of the included studies were investigated. Future studies should consider examining the completion rate of programs, as program attrition and nonuse attrition are persistent problems with digital interventions [160,161].

At the time of the literature search, there were no national standards for website developers and users to refer to when considering the selection of Australian web-based interventions for mental health. However, on November 30, 2020, the Australian Government released the National Safety and Quality Digital Mental Health Standards for health care providers to refer to when selecting digital media (including web-based programs) for the delivery of high-quality mental health care and suicide prevention (counseling or peer-based support) [162]. The purpose of the National Safety and Quality Digital Mental-Health Standards is to provide consumers and practitioners with information on which to base their selection of trustworthy resources and guide resource developers by providing quality standards. Familiarity and use of the standards, along with the findings from the present research, may help GPs and consumers select the most suitable web-based program for their needs. Although standards are now publicly available, an independent assessment tool to identify where a digital mental health service is meeting the standards or where it can improve is currently being developed and will not be available until late 2021. Similar reviews of this type in the future should include information on whether interventions have met these standards and whether this is a self- or independent assessment.

### Comparison With Prior Work

Overall, of the 52 programs, 9 (17%) addressed depression, 15 (29%) addressed anxiety, 13 (25%) addressed general mental well-being, and 13 (25%) addressed multiple issues. This is substantially lower than the 32 programs for depression and the 34 programs for anxiety identified by Renton et al [15] and Ashford et al [16], respectively. In part, this is because of the restriction of the current review to programs that were (1) free and (2) accessible to Australians, which excluded several currently available international programs such as Beating the Blues or Deprexis, which offer interventions for a fee or are not available to Australians. In addition, the results may highlight the difficulties consumers face in finding suitable programs through search engines [163]. Search engine results were based on the Australian version of Google; however, these results are likely to change over time, as do programs, and the same search conducted at a later date may yield different results. Our search terms were intentionally broad, and we did not require the terms to be used in conjunction with terms describing *therapy* or *self-help.* The purpose of broadening the search in this way was to mirror the type of search that a consumer is likely to perform (focused on the issue and not the intervention type).

### Conclusions

A total of 52 web-based programs and web-based programs with downloadable worksheets or resources programs are currently freely available to help Australians with the management of depression, anxiety, suicidal ideation, or general mental well-being. Careful attention is needed by clinicians and consumers to determine whether the interventions they refer to, or access, are evidence-based and considered acceptable by other users, given the varied levels of acceptability and impact. This review complements existing resources by providing website summaries and a clear comparison of website features to inform clinicians and consumers and assist in the selection of the most suitable program for the individual. It also identified important gaps in the availability of free web-based interventions in Australia (eg, for culturally and linguistically diverse populations and based on ACT), which may inform future research and program development initiatives.

## Appendix

Multimedia Appendix 1 Google search results.

Multimedia Appendix 2 Program developers and website links.

Multimedia Appendix 3 Program evaluation scores.

Multimedia Appendix 4 Types of research evaluations of eligible programs and summary of evidence.

## Abbreviations

ACT: acceptance and commitment therapy
CBT: cognitive behavioral therapy
CCI: Centre for Clinical Interventions
DBT: dialectical behavior therapy
eMHPrac: e-Mental Health in Practice
GP: general practitioner
LGBQ: lesbian, gay, bisexual, and queer
PRISMA-ScR: Preferred Reporting Items for Systematic Reviews and Meta-Analyses extension for Scoping Reviews
RCT: randomized controlled trial
