# Supplementary material for: Web-Based Interventions to Help Australian Adults Address Depression, Anxiety, Suicidal Ideation, and General Mental Well-being: Scoping Review
Source: JMIR Ment Health. 2022 Feb 8;9(2):e31018. doi: 10.2196/31018 (PMC8864526; doi:10.2196/31018)
Supplement: Multimedia Appendix 2 [file mental_v9i2e31018_app2.docx]

**Multimedia Appendix 2.**

Program developers and website links

| Program | Program developers | Website link |
| --- | --- | --- |
| BeyondNow | Beyond Blue | https://www.beyondblue.org.au/get-support/beyondnow-suicide-safety-planning/create-beyondnow-safety-plan |
| CCI^a^   - Depression - Health Anxiety - Panic - Social Anxiety Course - Tolerating Distress - Worry and Rumination | Department of Health, Western Australia | https://www.cci.health.wa.gov.au/Resources/Looking-After-Others |
| eCouch   - Anxiety & Worry - Bereavement & Loss - Depression - Divorce & Separation | Australian National University | https://ecouch.anu.edu.au/welcome |
| eCouch   - Social Anxiety | e-hub Health | https://ecouch.com.au/ |
| Evolution Health   - Overcoming Anxiety - Overcoming Depression - Grief & Loss - Managing Anxiety | Evolution Health Systems | https://evolutionhealth.care/index |
| ifarmwell | University of South Australia | https://www.ifarmwell.com.au/ |
| Living Life to the Full   - for Adults - for Farming Communities - with God - Enjoy Your Baby - Enjoy Your Bump | Five Areas Limited | https://llttf.com/ |
| Mental Health Online   - Depression Online - Generalized Anxiety Disorder - Made 4 Me - Panic STOP! - Social Anxiety Online | Swinburne University of Technology | https://www.mentalhealthonline.org.au/ |
| MindSpot   - Indigenous Wellbeing Course - Mood Mechanic Course - Wellbeing - Wellbeing Plus | MacQuarie University | https://mindspot.org.au/ |
| MoodGym | e-hub Health | https://moodgym.com.au/ |
| Mum2BMoodBooster | Parent-Infant Research Institute (Australia) and the Oregon Research Institute (United States) | https://www.mum2bmoodbooster.com/public/ |
| MumMoodBooster | Parent-Infant Research Institute (Australia) and the Oregon Research Institute (United States) | https://mummoodbooster.com/public/ |
| myCompass | Black Dog Institute | https://www.mycompass.org.au/ |
| My Digital Health   - iConsiderLife - Life Flex - Life Flex LGBQ^b^ | Federation University Australia | https://www.mydigitalhealth.org.au/ |
| OnTrack - Depression | Queensland University of Technology | https://www.ontrack.org.au/ |
| The Desk | The University of Queensland | https://www.thedesk.org.au/ |
| This Way Up   - Coping with Stress Course - The Depression Course - Health Anxiety Course - Mindfulness-based CBT^c^ Course - Mixed Depression and Anxiety Course - MUMentum Pregnancy - MUMentum Postnatal - Panic Attacks Course - Social Anxiety Course - Student Wellbeing Course - Worry Course (GAD^d^) | St Vincent’s Hospital and the University of New South Wales | https://thiswayup.org.au/ |
| Students Against Depression | The Charlie Waller Memorial Trust | https://www.studentsagainstdepression.org/ |

^a^CCI: Centre for Clinical Interventions; ^b^LGBQ: Lesbian, Gay, Bisexual & Queer; ^c^CBT: Cognitive Behavioral Therapy; ^d^GAD: Generalized Anxiety Disorder
