# Supplementary material for: Web-Based Interventions to Help Australian Adults Address Depression, Anxiety, Suicidal Ideation, and General Mental Well-being: Scoping Review
Source: JMIR Ment Health. 2022 Feb 8;9(2):e31018. doi: 10.2196/31018 (PMC8864526; doi:10.2196/31018)
Supplement: Multimedia Appendix 4 [file mental_v9i2e31018_app4.docx]

**Multimedia Appendix 4.**

Types of research evaluations of eligible programs and summary of evidence

| Program | Type of Research Evaluation | Studies Outcome of Evaluation |
| --- | --- | --- |
| BeyondNow | - Feasibility and effectiveness study [42] | The program observed a reduction in participant severity and intensity of suicide ideation, and suicide-related coping increased significantly. No significant changes were observed in suicide resilience [42]. |
| CCI^a^ – Depression | Website: not specified; Beacon^b^: not reviewed | - |
| CCI^a^ – Health Anxiety | - Preliminary pre-post study [43] | The program improved patients’ worry, generalized anxiety disorder symptoms and metacognitions [43]. |
| CCI^a^ – Panic | Website: not specified; Beacon^b^: not reviewed | - |
| CCI^a^ – Social Anxiety Course | Website: not specified; Beacon^b^: not reviewed | - |
| CCI^a^ – Tolerating Distress | Website: not specified; Beacon^b^: not reviewed | - |
| CCI^a^ – Worry and Rumination | - Preliminary pre-post study [43] | All 3 participants achieved clinically significant improvement on measures of worry, GAD^c^ symptomatology, and metacognitions. None of the participants met the diagnostic criteria for GAD^c^ at the completion of the study, while they did at the beginning [43]. |
| eCouch – Anxiety & Worry | Adapted from MoodGym  - RCT^d^ [44, 45] | RCT^d^ results were mixed. In one RCT^d^, the program reduced generalized anxiety disorder symptoms relative to an attention control condition immediately post-intervention, but not at 6 or 12 months follow up [45]. However, in another RCT^d^ the intervention did not reduce generalized anxiety disorder symptoms compared to the control condition, however, worry and anxiety sensitivity were reduced in the group receiving email reminders at post-test and anxiety sensitivity was reduced in both email and telephone reminder groups at 6 months. In addition, the program (with email reminders) had an impact on secondary outcomes such as depression [44]. |
| eCouch – Bereavement & Loss | Adapted from MoodGym | - |
| eCouch – Depression | Adapted from MoodGym  - RCT^d^ [46-50] | The eCouch Depression Program showed a significant reduction in depressive symptoms at post-test and follow-up for both the CBT^e^ and IPT^f^ components of the program and were noninferior to MoodGym [46]. Moreover, the program may generate secondary benefits, and in particular may increase personal empowerment, and improve self-esteem and quality of life [47] and may be more beneficial to people aged 25 and older [50]. Further, the program produced a small improvement in depressive symptoms, adherence, and some health behaviors such as increased physical activity in people aged over 45 years with cardiovascular disease [48, 49]. |
| eCouch – Divorce & Separation | Adapted from MoodGym | - |
| eCouch – Social Anxiety | Adapted from MoodGym  - Two-group RCT^d^ and to assess Effectiveness & Cost-effectiveness [12]  - Pre- and post with control group and randomized [51] | Participants reported significantly reduced levels of social anxiety, trait anxiety, and depression and improved attentional control [51]. For people with social anxiety symptoms who are not receiving other forms of help, results suggested that the use of an online self-help tool based on cognitive behavioral principles can provide a small improvement in social anxiety symptoms compared with no intervention [12]. The economic evaluation demonstrated cost-effectiveness with a small health status benefit and a reduction in health service utilization [12]. |
| Evolution Health – Overcoming Anxiety | - Pre-post longitudinal study [52]  - Pilot RCT^d^ [53]  - User characteristics [54] | Panic attack frequency and severity decreased post-program compared to pre-program [52]. User characteristics and program elements for all Evolution Health programs have been examined in further studies [53, 54]. |
| Evolution Health – Overcoming Depression | Website: not specified; Beacon^b^: not reviewed | - |
| Evolution Health – Grief & Loss | Website: not specified; Beacon^b^: not reviewed | - |
| Evolution Health – Managing Anxiety | - Pre-post longitudinal study [52] | Panic attack frequency and severity decreased post-program compared to pre-program [52]. |
| ifarmwell | Website: currently being evaluated; Beacon^b^: not reviewed | - |
| Living Life to the Full for adults (LLTTF) | - Comparative clinical feasibility study [55]  - Feasibility [56] | The program produced significant clinical benefit for adults with mild to moderate depression and anxiety, thus indicating an improvement in mental health. There was no significant difference in the level of improvement found with each of the three self-help CBT^e^ approaches (LLTTF online intervention, a paid online intervention, and a workbook-based intervention) [55]. In another study [56], significant improvements in mental health scores were found after exposure to the intervention and sig. reduction in numbers reaching clinical threshold limits for mental health difficulties. |
| Living Life to the Full for Farming Communities | Website: not specified; Beacon^b^: not reviewed | - |
| Living Life to the Full with God | Website: not specified; Beacon^b^: not reviewed | - |
| Living Life to the Full - Enjoy Your Baby | Website: not specified; Beacon^b^: not reviewed | - |
| Living Life to the Full – Enjoy Your Bump | Website: not specified; Beacon^b^: not reviewed | - |
| Mental Health Online – Depression Online | - Uncontrolled pre-post treatment study [57, 58] | The program significantly reduced levels of depression for those who completed the depression program [57, 58] and those who reported greater pre-treatment depression symptomatology showed greater reduction in symptoms at post-test. Participating in the intervention also decreased anxiety, stress, and improved quality of life, across a combined sample of participants completing the depression, GAD^c^ or panic programs [57]. Program satisfaction was high [57]. |
| Mental Health Online – Generalized Anxiety Disorder | - Uncontrolled pre-post treatment study [57]  - Pre-post treatment [59]  - Pre- to posttreatment quasi-experimental (participant choice) [60] | The program significantly improved anxiety [57, 59, 60], confidence levels in managing one’s own mental health, quality of life, general psychological distress [59, 60]. In addition, those who reported greater pre-treatment depression symptomatology showed greater reduction in symptoms at post-test. In a combined sample of participants completing the depression, GAD^c^ or panic programs, levels of depression, stress and quality of life also improved [57]. Program satisfaction was high [57]. Pre-treatment attrition and treatment withdrawal has also been examined [59]. |
| Mental Health Online – Made 4 Me | Website: not specified; Beacon^b^: not reviewed | - |
| Mental Health Online – Panic STOP! | - Uncontrolled pre-post treatment study [57]  - Pre-post treatment [59]  - Participant choice quasi-experimental trial [60] | The program significantly improved panic symptoms [57], panic disorder severity ratings, confidence levels in managing one’s own mental health, and general psychological distress [59, 60]. No difference was observed in quality of life [60]. In a combined sample of participants completing the depression, GAD^c^ or panic programs, levels of depression, stress and quality of life also improved [57]. Program satisfaction was high [57]. |
| Mental Health Online – Social Anxiety Online | - Pre-post treatment study [59]  - Participant choice quasi-experimental trial [60] | The program significantly improved social anxiety disorder severity ratings, confidence levels in managing one’s own mental health, and participants’ quality of life [59, 60]. Findings for general psychological distress were mixed. |
| MindSpot – Indigenous Wellbeing Course | - Prospective uncontrolled observational cohort study [61] | The program was effective in treating anxiety and depression in Indigenous Australians, and outcomes were similar to those of non-Indigenous patients [61]. |
| MindSpot – Mood Mechanic Course | - Single-arm, open trial [62]  - RCT^d^ [63] | The program was effective in reducing symptoms of anxiety and depression in both the clinical-guided and self-guided groups. Significant improvements were also observed in general psychological distress, satisfaction with life and disability [62, 63]. |
| MindSpot – Wellbeing | - Cost-effectiveness [64]  - Feasibility trial [65]  - RCT^d^ [66-73]  - 12-month follow-up RCT^d^ [62]  - Single group open trial [74] | Feasibility and noncontrolled studies showed that the program decreased anxiety and depression [65], and also decreased psychological distress and disability for people with anxiety [65]. In RCTs^d^, the program reduced elevated symptoms of anxiety and depression when compared to a control [67], reduced anxiety in a sample of people diagnosed with generalized anxiety disorder, panic disorder or social phobia [71], and reduced panic symptoms, depression, generalized anxiety, social anxiety and psychological distress, for people diagnosed with panic disorder [70], generalized anxiety disorder [69], social anxiety disorder [68], and major depressive disorder [66]. The program reduced depression and anxiety symptoms for adults with symptoms of depression, generalized anxiety disorder, panic disorder or social phobia [72] and in a later trial [67], and this was sustained at 12 months follow-up [62]. Overall, the program was equally effective when compared to a disorder-specific program [66, 68-70], or to a waitlist control [67], was highly acceptable to users [65, 67], and was cost-effective in comparison to usual care [64]. In addition, adherence has also been examined [62, 67]. Further, ddifferent versions of the program have also been evaluated. For example, reduced symptoms of anxiety and depression were found in people after completing a brief version [74] and in people with co-morbid conditions [73] and when compared with a group receiving coach support [72]. |
| MindSpot – Wellbeing Plus | - Cost-effectiveness [75, 76]  - Feasibility study [75, 77]  - Implementation [78]  - RCT^d^ [75, 76, 79, 80] | The program was effective at reducing anxiety and depression in older adults [75, 77] and in older adults with multimorbidity [80]. In RCTs^d^, the program was shown to be equally acceptable and as effective as clinician-guided internet-delivered CBT^e^ [79], and more effective than a waitlist control condition [75, 76], in improving symptoms of depression and anxiety among adults with depression or anxiety. The program is cost-effective [75, 76] and has been shown to be as effective and acceptable in clinical settings as it is in randomized controlled trials [78]. |
| MoodGym | - Acceptability study [81]  - Implementation [82]  - Follow-up outcome analysis [83]  - Program usage [84]  - RCTs^d^ [10,17, 81, 82, 85-99]  - School/class trials [100, 101]  - Compliance [102] | Overall, cognitive behavior therapy and psychoeducation delivered via the internet are effective in reducing symptoms of anxiety and depression and negative thoughts and improve mental well-being in both adolescents and adults [10, 17, 81, 82, 85-91, 94-96, 98, 100-102]. Furthermore, the program can be used effectively in the community [82] and is considered to be at least as acceptable as seeing a professional about mental health issues [81]. However, no substantial to only small improvements in depression outcomes were observed when comparing usual GP^g^ care to GP^g^ care plus the program [93, 99]. Moreover, the program does not reduce depression or anxiety for patients who are waiting for public mental health service interventions [97]. |
| Mum2BMoodBooster | Website: currently being evaluated; Beacon^b^: not reviewed | - |
| MumMoodBooster | - Acceptability study [103, 104]  - Feasibility study [104]  - RCT^d^ [105] | New mothers scored the program as having high usability and reported high program satisfaction [103, 104]. Post-program completion, participant scores were lower on measures of depression, believability of thoughts, and higher on measures of behavioral activation for depression, self-efficacy, and parenting sense of competence, for mild to moderately severely depressed women [104]. No change was reported for relationship satisfaction [104]. In an RCT^d^ of women with a clinical diagnosis of postnatal depression, the MumMoodBooster program was found to be more effective at improving depression and behavioral activation compared to usual care [105]. The study also reported an improvement in automatic thinking and parenting sense of competence, with medium-large effect sizes, although neither of these results were significant. The intervention was found to be more effective at reducing stress at 9 weeks than usual care, although this was not significant at 12 weeks. There was no change in relationship satisfaction or anxiety [105]. |
| myCompass | - Feasibility study [106]  - Open trial [107]  - RCT^d^ [108-110] | The myCompass program is effective and showed significant improvements in mental health self-efficacy and symptoms of depression, anxiety, and stress as well as in work and social functioning [107-109]. The program also showed high satisfaction among people with diabetes, and significantly improved symptoms of depression, anxiety, functioning and diabetes related stress in this population [106, 110]. |
| My Digital Health – iConsiderLife | Website: currently being evaluated; Beacon^b^: not reviewed | - |
| My Digital Health – Life Flex | Website: currently being evaluated; Beacon^b^: not reviewed | - |
| My Digital Health – Life Flex LGBQ | Website: currently being evaluated; Beacon^b^: not reviewed | - |
| OnTrack - Depression | Website: not specified; Beacon^b^: not reviewed | - |
| The Desk | Website: not specified; Beacon^b^: not reviewed | - |
| This Way Up – Coping with Stress Course | Website: not specified; Beacon^b^: not reviewed | - |
| This Way Up – The Depression Course | - Non-randomized comparison study [111]  - Open trial [112-117]  - RCT^d^ [111, 118-124]  - Adherence [125] | Trials have indicated that the program reduced depression, anxiety, distress, and disability [111-114], and suicidal ideation [115, 116]. RCTs^d^ have identified significant reductions in depression compared to waitlist controls [118, 121] or delayed treatment controls [119], and have found that the program is equally efficacious when delivered via internet or mobile application [122]. A recent RCT^d^ found that the iCBT program was more effective at reducing depressive symptoms and distress, compared to a monitored attention control, for participants with major depressive disorder [120]. In another recent study [124], researchers allocated patients in a waiting room to MyCompass (mild symptoms), This Way Up (moderate symptoms) or nurse-led collaborative care (severe symptoms), compared to ‘usual care’. They found matching the severity of symptoms to a particular intervention improved symptomatology at 3 months compared with usual care. Satisfaction with the program was high [118]. Studies have also examined the effectiveness of the program among groups with co-morbid conditions e.g., [123]). Adherence has also been examined [125]. |
| This Way Up – Health Anxiety Course | - Open trial [126, 127]  - RCT^d^ [128] | An open pilot trial found that the program reduced health anxiety, depression, distress, anxiety, disability, body vigilance, worry behaviors and dysfunctional cognitions [126]. Another open trial recently found that the program decreased health anxiety, depression, and distress [127]. An RCT^d^ found that reductions in health anxiety, depression, generalized anxiety, worry behaviors and dysfunctional cognitions were greater in the iCBT^e^ group compared to a psychoeducation control group [128]. Satisfaction with the program was high [126, 128]. Adherence has also been examined [126, 127]. |
| This Way Up –Mindfulness-based CBT Course | - Open trial [129]  - RCT^d^ [130] | The program decreased distress, anxiety, depression, and functional impairment [129, 130], and was superior to a treatment as usual control condition [130]. In a pilot study the program also decreased worry, rumination, experiential avoidance and emotion regulation, and improved trait mindfulness and well-being ratings [129]. Satisfaction with the program was high [129]. |
| This Way Up – Mixed Depression and Anxiety Course | - Open trial [74, 111, 131-133]  - RCT^d^ [72, 131, 134] | RCTs^d^ have shown that the program significantly reduces anxiety and depression compared to a waitlist control group [72, 131, 134]. Open trials have demonstrated a reduction in depression, anxiety, disability [131, 133], social and panic disorder symptoms, and suicidal ideation [131], the effectiveness and acceptability of brief versions of the program [87, 147], and the effectiveness of the program compared to disorder-specific iCBT^e^ [111]. Adherence has also been examined e.g., [111, 131, 132, 134]. |
| This Way Up –MUMentum Pregnancy | - RCT^d^ [135] | The program reduced anxiety and psychological distress and was superior to a treatment as usual control. Participants in both groups reported reduced depression and improved psychological quality of life over the course of the trial. Program satisfaction was high [135]. |
| This Way Up –MUMentum Postnatal | - RCT^d^ [136] | The program reduced anxiety, depression, psychological distress, and improved maternal bonding, parenting confidence and psychological and social quality of life, and was superior to a treatment as usual control. No between group differences were observed for parenting confidence, physical or environmental quality of life. Program satisfaction was high [136]. |
| This Way Up –Panic Attacks Course | - Open trial [137]  - RCT^d^ [71, 72, 138-140] | A pilot open trial showed a reduction in the symptoms of panic disorder and agoraphobia [137]. RCTs^d^ have shown that, compared to a waitlist control, the program reduced symptoms of panic [139, 140] distress, disability, and depression [140]. Participants randomized to internet-based CBT treatment for generalized anxiety disorder, panic disorder or social phobia, demonstrated greater improvements in outcomes such as work and social adjustment, anxiety, and social phobia, compared to a waitlist control group [71, 72, 138]. Adapted versions of the program and adherence have also been evaluated and examined. |
| This Way Up –Social Anxiety Course | - Open trial [141]  - RCT^d^ [71, 72, 138, 142-146] | RCTs^d^ have shown that the program decreases symptoms of social phobia, compared to waitlist controls [142, 143], with a clinician-assisted version of the program showing the greatest improvements compared to both a self-help version and waitlist control [144]. The program is equally effective as face-to-face CBT in reducing symptoms of social phobia and disability [145]. RCTs^d^ have also found a reduction in social phobia symptoms, and comorbid symptoms of depression and anxiety, among groups with and without these comorbidities [146]. Additionally, participants randomized to internet based CBT^e^ treatment for generalized anxiety disorder, panic disorder or social phobia, demonstrated greater improvements in outcomes such as work and social adjustment, anxiety, and social phobia, compared to a waitlist control group [71, 72, 138] . Different versions of the program have also been evaluated. Program acceptability is high e.g. [143]. Adherence has also been examined. |
| This Way Up –Student Wellbeing Course | Website: not specified; Beacon^b^: not reviewed | - |
| This Way Up – Worry Course (GAD^c^) | - Non-randomized comparison study [111]  - Open trial [112, 133, 147, 148]  - RCT^d^ [71, 72, 138, 149-151] | Trials have indicated that the program significantly reduced anxiety, distress, disability, and depression for most users [111, 112, 133, 147, 148]. Compared to delayed treatment or waitlist control groups, participants using the program reported lower worry and generalized anxiety symptoms [149-151] and depression [150]. Participants randomized to internet based CBT^e^ treatment for generalized anxiety disorder, panic disorder or social phobia, demonstrated greater improvements in outcomes such as work and social adjustment, anxiety, and social phobia, compared to a waitlist control group [71, 72, 138]. Adherence has also been examined e.g., [111, 133, 148]. |
| Students Against Depression | Website: currently being evaluated; Beacon^b^: not reviewed | - |

^a^CCI: Centre for Clinical Interventions.
^b^Beacon: Australian clinical online platform that describes different Web-based self-help treatment programs.
^c^GAD: Generalized Anxiety Disorder.
^d^RCT: randomized controlled trial.
^e^CBT: Cognitive Behavior Therapy.
^f^IPT: Interpersonal Therapy.
^g^GP: General Practitioner.
